# The Role of Autophagy in Brain Tumors

**DOI:** 10.3390/cancers15194802

**Published:** 2023-09-29

**Authors:** Gabriella D’Orazi

**Affiliations:** 1 Unit of Cellular Networks, Department of Research, IRCCS Regina Elena Cancer National Institute, 00144 Rome, Italy; gdorazi@unich.it; 2 Department of Neurosciences, Imaging and Clinical Sciences, University G. D’Annunzio, 00131 Chieti, Italy

Primary and metastatic brain tumors are among the most threatening diseases worldwide [1,2]. Even improved surgical interventions often cannot completely eradicate brain tumors that progress and metastasize, reducing the patient’s chance of survival. Current therapeutic options, such as radio- and chemotherapies, as well as immunotherapy, are also ineffective because brain tumors are highly resistant to therapies. Autophagy is a highly conserved cellular homeostatic process that maintains cellular homeostasis through the degradation, elimination, and recycling of damaged substrates, such as organelles, macromolecules, and misfolded proteins [3]. However, its deregulation is associated with several pathological processes, including cancer [4]. It has been demonstrated that autophagy has a double role, both as a tumor promoter and as a suppressor. It promotes cancer initiation and survival through the recycling of intracellular substrates in order to sustain tumor metabolism, contributing to the acquisition of resistance to treatments; and it inhibits cancer progression by removing damaged proteins and organelles, in order to protect cells from reactive oxygen species (ROS), inflammation, necrosis, and other causes of genomic instability. Therefore, autophagy manipulation could be a novel anticancer strategy exploited to improve the outcome of cancer treatments [5], including brain tumors, as shown in this series of five articles (one review and four articles).

The review by Pizzimenti et al. summarizes the molecular mechanisms as well as the regulatory pathways of autophagy, mainly addressing their involvement in human astrocytic neoplasms. The role of autophagy both as a tumor promoter or tumor suppressor is explained. The review also discusses the relationships between autophagy, the tumor immune microenvironment, and glioma stem cells. Finally, an excursus concerning autophagy-targeting agents is included along with the ongoing clinical trials targeting autophagy in glioma. Novel methods such as clustered regularly interspaced short palindromic repeats (CRISPRs)—CRISPR-associated protein (Cas9) genome editing and/or application of miRNAs and high-throughput technologies are underlined as key technologies to identify and understand autophagy targets in gliomas, in order to obtain additional information for the better treatment and management of therapy-resistant patients. Thus, it is suggested that this modern perspective could help in the selection of patients with gliomas that are most likely to respond to autophagy inhibition therapy, but also to identify patients resistant to treatment [6].

In the study by McCartin et al., the authors developed a new chemotherapeutic compound able to target cancer stem cells (CSCs) that are often responsible of tumor resistance to therapies. The new compound was developed in order to improve the biocompatibility of N-heterocyclic carbene (NHC) as a platinum (Pt)-stabilizing ligand, which has demonstrated cytotoxic activities superior to cisplatin or related compounds, along with high stability. However, such compounds still present some drawbacks such as their biocompatibility. In the study, the authors developed a new platinum-based polyethylenimine (PEI) polymer–drug conjugate (PDC), a long polymer molecule with the chemical characteristics able to enhance its anti-cancer properties. Thus, the compound induced necrotic cell death instead of apoptosis, potentially bypassing the resistance to apoptosis of CSCs. The cell death was accompanied by an induction of a protective autophagy response. The implication of the autophagic pathway in the compound’s mechanism of action is suggested as a highly promising target in its application against CSCs, as it is a pathway for which balance is key in the maintenance of their metastatic and drug-resistant phenotype [7].

Epigenetic deregulation by histone modifications leads to oncogene upregulation and tumor suppressor gene downregulation in numerous cancer types including glioblastoma multiforme (GBM), a grade IV, highly malignant brain tumor mostly common in adults. In the study by Chang et al., the authors evaluated class I/II histone deacetylases inhibitors (HDACis) valproate and sodium phenylbutyrate (PBA) for their antitumor activities on GBM cell lines. They found that LMK235, a selective HDACi, induces GBM cell death via autophagy activation. Further validation showed that LMK235 significantly reduces the mRNA and protein expression of SCNN1A. As a proof of principle, SCNN1A gene silencing reduces cell viability that was rescued by the autophagy inhibitor bafilomycin A1. Although the study presents some limitations, such as the dose of LMK235 used, a multitarget and multifunctional compound dependent on its dosage and cell types, the authors underline its potential use in the treatment of GBM. Moreover, they suggest that SCNN1A plays a role in LMK235-induced autophagy and cell death in GBM cells, becoming an alternative therapeutic target for GBM [8].

To search for new therapeutic approaches is urgently needed to improve the outcome of patients with GBM. In this regard, Filippone et al. attempted to evaluate sodium propionate (SP), a short-chain fatty acid (SCFA), as an anticancer agent. Interesting pieces of evidence indicate the SFCA’s role in reduction of tumor growth through inhibition of the NF-κB inflammatory and histone deacetylase pathways. In addition, sodium butyrate (SB) is able to modulate the immune system, oxidative stress, interleukins (ILs)/cytokines release, and inflammation in many in vitro and in vivo studies. In this in vitro and in vivo study, the authors found that SP promotes apoptosis and autophagy through a PPAR-γ-dependent mechanism, reducing GBM tumor progression. Knockdown of PPAR-γ sensitized GBM cells and blocked the SP effect, suggesting that the PPAR- γ/SCFAs axis could be a potential target for the management of GBM [9].

ONX-0914 (PR957) is a proteasome subunit beta type-8 (PSMB8)-selective inhibitor. Previous studies have shown that inhibiting PSMB8 expression in a glioblastoma reduces tumor progression and angiogenesis. In the study by Chang et al., the authors found that ONX-0914 treatment inhibited survival of several GBM cell lines and in an orthotopic animal model. ONX-0914 induced apoptosis in a p53-dependent manner and autophagy in GBM cells. In addition, temozolomide (TMZ) combined with ONX-0914 inhibited in vivo GBM growth. Although the study presents some limitations, the results provide a strong rationale for testing PSMB8 inhibitors in patient samples and ultimately in clinical trials in GBM [10].

We hope readers enjoy this series of unique articles aiming at unveiling the role of autophagy in brain tumor progression and response to therapies. We hope that this dissertation will help to understand the potential impact of autophagy manipulation in brain tumors treatment and encourage the development of new anticancer strategies against this type of cancer.
